# Effects of Sn Addition and Fe Content Adjustment on the Decolorization Performance of Fe-Si-B Amorphous Alloys for Azo Dyes

**DOI:** 10.3390/ma18102240

**Published:** 2025-05-12

**Authors:** Jing Wei, Zhigang Zheng, Zhaoguo Qiu, Wanghui Xu, Meng Xiao, Dechang Zeng

**Affiliations:** 1 School of Shipping and Maritime Studies, Guangzhou Maritime University, Guangzhou 510725, China; 2 School of Materials Science and Engineering, South China University of Technology, Guangzhou 510640, China; mszgzheng@scut.edu.cn (Z.Z.); zgqiu@scut.edu.cn (Z.Q.); medczeng@scut.edu.cn (D.Z.); 3 Zhongshan R&D Center for Materials Surface and Thin Films Technology of the South China University of Technology, Gent Materials Surface Technology (Guangzhou) Co., Ltd., Zhongshan 528437, China; 4 School of Intelligent Manufacturing, Guangzhou Maritime University, Guangzhou 510725, China; xuwanghui@126.com; 5 School of Chemistry and Materials Science, Guangdong University of Education, Guangzhou 510640, China

**Keywords:** Fe-Si-B amorphous alloys, decolorization performance, azo dyes, Sn addition, Fe content

## Abstract

This study compares the effects of adding Mo, Cu, and Sn elements on the decolorization performance of Fe_77_Si_13_B_9_M_1_ (M = Mo, Cu, or Sn) amorphous alloys. After the addition of Cu and Sn elements, the Fe-Si-B amorphous alloys generate three-dimensional (3D) petal-like nanostructured corrosion products during the decolorization process. These petal-like nanostructures possess a high specific surface area and excellent adsorption capacity, thereby effectively promoting the decolorization of dyes. Furthermore, the influence of Fe content variation on the decolorization performance of Fe_77+x_Si_13−x_B_9_Cu_1_ (x = 0, 2, or 4) and Fe_77+x_Si_13−x_B_9_Sn_1_ (x = 0, 2, or 4) alloys was investigated. The glass-forming ability of Fe_77+x_Si_13−x_B_9_Cu_1_ alloys decreases with increasing Fe content, leading to the precipitation of α-Fe crystalline phases starting from Fe_79_Si_11_B_9_Cu_1_. As the crystallinity increases, the decolorization performance of the alloys gradually deteriorates. In contrast, the Fe_77+x_Si_13−x_B_9_Sn_1_ alloys maintain their amorphous structure even with increasing Fe content, and their decolorization performance for Orange II improves accordingly. The high decolorization efficiency of FeSiBSn amorphous alloys for Orange II can be attributed to their unique self-refreshing properties.

## 1. Introduction

Azo dyes, as predominant pollutants in textile, printing, and leather industries, are characterized by high chromaticity, toxicity, and recalcitrance, posing severe threats to ecosystems and human health upon wastewater discharge [1,2,3]. Traditional treatment methods include biological, physical adsorption, and chemical oxidation approaches. The biological method primarily utilizes microbial enzymes to break down the unsaturated bonds and chromophore groups within dye molecules, converting large molecules into smaller ones, ultimately achieving complete degradation of azo dyes [4,5]. However, the biological method exhibits poor adaptability to high-concentration dye wastewater and may generate intermediate products that are biologically toxic. The physical method mainly relies on differences in the physical properties of various substances to separate or collect them. Common physical methods include adsorption, membrane separation, magnetic separation, and extraction [6,7]. While physical methods are generally straightforward to operate, they are associated with high costs. The chemical method primarily involves chemical reactions with chromophore groups to cleave these groups, transforming large organic molecules into smaller ones, which are further degraded, ultimately leading to the mineralization of azo dyes. This approach includes methods such as electrochemical oxidation, photocatalytic oxidation, ozone oxidation, Fenton oxidation, and zero-valent iron reduction [8,9]. Oxidation methods typically require external inputs of electricity, light, or chemical reagents, which results in higher operational costs and substantial sludge production. Consequently, the development of efficient, stable, and environmentally benign treatment technologies has emerged as a research priority.

Zero-valent iron (ZVI, Fe^0^) reduction is one of the commonly used chemical methods for the degradation of azo dyes. The main reasons are its high production yield, low cost, and non-toxic, harmless nature to biological organisms. Fe-based amorphous alloys contain a large amount of metastable Fe^0^, which imparts high reactivity and energy. At the same time, the corrosion resistance of Fe-based amorphous alloys is superior to that of conventional crystalline ZVI, addressing the issue of easy passivation in crystalline ZVI [10,11,12]. Studies have shown that their efficiency in degrading azo dyes can be tens to hundreds of times greater than that of commercial iron powders [13]. This superior performance originates from abundant active sites and anti-passivation capabilities on the amorphous alloy surface, where the metastable structure enables continuous release of active Fe atoms, facilitating reduction reactions and free radical generation [14,15,16].

It is worth noting that, under normal circumstances, researchers aim to alter certain properties of the original alloy by introducing trace amounts of other metals or metalloid elements [17,18,19,20]. This approach is intended to enhance the degradation performance of azo dyes. When coupled with less-active metals, Fe-based amorphous alloys exhibit accelerated Fe^0^ oxidation rates via galvanic cell effects, thereby amplifying reactivity [21,22,23,24]. Yao et al. developed Fe-AR-NCI, a Fe-based amorphous ribbon with nanoscale compositional heterogeneity, by incorporating Cu into FeSiB alloy [25]. This modification achieved exceptional degradation performance for Orange II, demonstrating a half-life (t_1/2_) of 5 min—80-fold faster than ZVI-P300 (t_1/2_: 160 min) under identical conditions.

The decolorization mechanism involves two primary reactions (Equations (1) and (2)): Fe^0^ atoms and adsorbed Fe^2+^ donate electrons to reduce the azo bond (−N=N−), cleaving the conjugated chromophore system. This underscores the necessity to investigate how enhanced Fe content influences decolorization performance [26,27].

(1) $$2{\mathrm{Fe}}^{0}+R-N=N-R’+4H^{+}\to 2{\mathrm{Fe}}^{2+}+R-{\mathrm{NH}}_{2}+R’-{\mathrm{NH}}_{2}$$

(2) $$4{\mathrm{Fe}}^{2+}\;(\mathrm{adsorbed})+R-N=N-R’+4H^{+}\to 4{\mathrm{Fe}}^{3+}+R-{\mathrm{NH}}_{2}+R’-{\mathrm{NH}}_{2}$$

The standard electrode potential of Fe^2+^/Fe^0^ is −0.447 V. Therefore, this study intends to introduce small amounts of elements with electrode potentials higher than that of Fe^0^, forming a galvanic cell structure to accelerate the degradation of azo dyes. In addition, Mo elements can stimulate the reaction activity of Fe^0^ by coordinating with surface electronic transfer [28], while Sn elements can enhance the amorphous formation ability [29]. The effects of adding Mo (−0.2 V, Mo^3+^/Mo^0^), Cu (+0.345 V, Cu^2+^/Cu^0^), and Sn (−0.138 V, Sn^2+^/Sn^0^) elements on the decolorization performance of Fe_77_Si_13_B_9_M_1_ (M = Mo, Cu, or Sn) amorphous alloys were investigated. Finally, the influence of Fe content variation on the decolorization performance of Fe_77+x_Si_13−x_B_9_Cu_1_ (x = 0, 2, or 4) and Fe_77+x_Si_13−x_B_9_Sn_1_ (x = 0, 2, or 4) alloys was explored, and the mechanism of efficient decolorization by FeSiBSn amorphous alloys was clarified.

## 2. Experimental

All raw materials used in this study were of industrial-grade purity: iron (Fe, 99.99 wt.%), silicon (Si, 99.99 wt.%), copper (Cu, 99.99 wt.%), tin (Sn, 99.99 wt.%), molybdenum (Mo, 99.99 wt.%), and boron (B, 99.99 wt.%). The alloys were prepared with nominal compositions of Fe_78_Si_13_B_9_, Fe_77_Si_13_B_9_M_1_ (M = Mo, Cu, or Sn), Fe_77+x_Si_13−x_B_9_Cu_1_ (x = 0, 2, or 4), and Fe_77+x_Si_13−x_B_9_Sn_1_ (x = 0, 2, or 4). Ingot melting was performed in a vacuum arc melting furnace under an argon atmosphere. The alloy ingot was inductively melted under an argon atmosphere until it reached a molten state. After maintaining the molten state for 3 s, the melt was ejected by gas flow onto a rapidly rotating copper wheel, with a wheel speed of 55 m/s, resulting in the formation of a ribbon-shaped amorphous alloy. The resulting ribbons exhibited a thickness of approximately 10 μm and were cut into 10~15 mm ribbons for subsequent experiments. Orange II (C_1__6_H_1__1_N_2_Na_4_S), employed as the target pollutant, was purchased from Sigma-Aldrich (Bayswater, Australia).

This experiment utilizes X-ray diffraction (XRD, X′Pert Powder, PANalytical, Netherlands) to identify the phase structure of the surface of the ribbon, employing Cu target Kα radiation with a wavelength of λ = 1.54056 Å. The surface morphology of the samples is observed and analyzed using a thermal field emission scanning electron microscope (SEM, NOVA NANOSEM 430, FEI, Peabody, MA, USA). Additionally, an Energy Dispersive Spectrometer (EDS) attached to the SEM is used to perform compositional analysis of the elements on the surface of the sample.

The dye decolorization experiments were conducted as follows: First, a 250 mL solution of Orange II azo dye (40 mg/L) was prepared in a 500 mL beaker. The beaker was placed in a thermostatic water bath maintained at a set temperature. The pH of the solution was adjusted to the desired value using 1 mol/L hydrochloric acid (HCl) and 1 mol/L sodium hydroxide (NaOH). Pre-weighed ribbons were added to the beaker, and mechanical stirring was initiated at a rate of 350 rpm. During the degradation process, 5 mL aliquots of the solution were periodically sampled using a disposable syringe and filtered through a 0.45-μm membrane to remove impurities. The filtered solution was immediately analyzed for absorbance using a UV-Vis spectrophotometer (UV–vis, TU-1900, Persee, Beijing, China).

## 3. Results and Discussion

**Figure 1:**
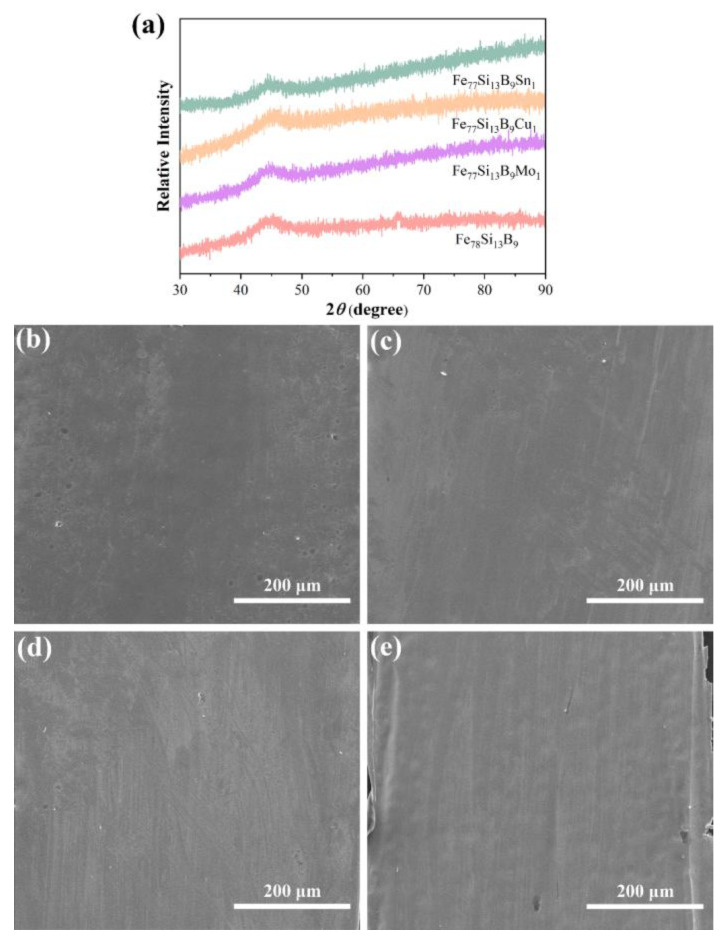
(**a**) XRD spectra of the prepared Fe_78_Si_13_B_9_ and Fe_77_Si_13_B_9_M_1_ (M = Mo, Cu, or Sn) amorphous alloys; (**b**) SEM image of Fe_78_Si_13_B_9_; and (**c**–**e**) SEM images of Fe_77_Si_13_B_9_M_1_ (M = Mo, Cu, or Sn) amorphous alloys.

Figure 1 presents the XRD patterns of the as-prepared Fe_78_Si_13_B_9_ and Fe_77_Si_13_B_9_M_1_ (M = Mo, Cu, or Sn) amorphous alloys. As observed, all alloy ribbons exhibit a broad diffuse scattering peak centered at approximately 45°, with no sharp crystalline peaks corresponding to long-range atomic order. This characteristic is attributed to the short-range ordered but long-range disordered atomic arrangement typical of amorphous structures, resulting in diffuse scattering patterns [30]. These findings confirm the fully amorphous nature of the prepared samples. Figure 1b–e show the SEM images of the Fe_78_Si_13_B_9_ and Fe_77_Si_13_B_9_M_1_ (M = Mo, Cu, or Sn) amorphous alloys. The images reveal smooth surfaces of the alloy ribbons without noticeable defects.

**Figure 2:**
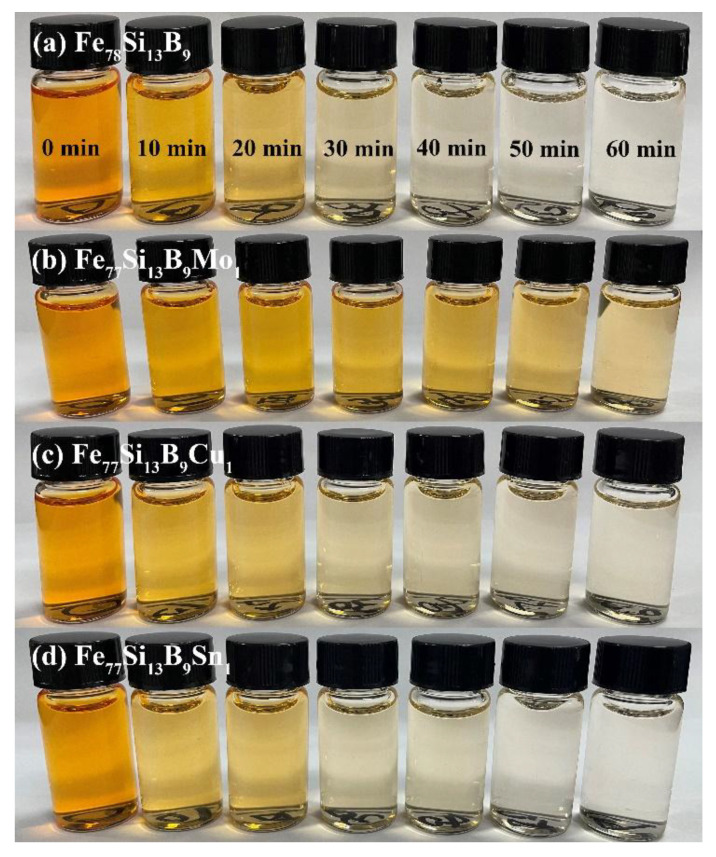
Color changes of Orange II solutions during the redox reactions of (**a**) Fe_78_Si_13_B_9_ and (**b**–**d**) Fe_77_Si_13_B_9_M_1_ (M = Mo, Cu, or Sn) amorphous ribbons.

Figure 2 illustrate the time-dependent color changes of Orange II solutions degraded using Fe_78_Si_13_B_9_ and Fe_77_Si_13_B_9_M_1_ (M = Mo, Cu, or Sn) amorphous ribbons under the following reaction conditions: temperature *T* = 35 °C, initial pH_0_ = 7, ribbon dosage = 2 g/L, and initial AO II concentration *C*_Orange II_ = 40 mg/L. As shown in Figure 2, the AO II solutions degraded by Fe_78_Si_13_B_9_, Fe_7__7_Si_1__3_B_9_Cu_1_, and Fe_77_Si_13_B_9_Sn_1_ ribbons became nearly colorless after 40 min, indicating almost complete decolorization. In contrast, Figure 2b reveals that the Orange II solution treated with Fe_77_Si_13_B_9_Mo_1_ ribbons still exhibited noticeable coloration even after 60 min, suggesting incomplete decolorization at this time point. Notably, during the initial 10 min of the decolorization process, the Fe_7__7_Si_1__3_B_9_Cu_1_ and Fe_77_Si_13_B_9_Sn_1_ ribbons demonstrated superior decolorization efficiency compared to the Fe_78_Si_13_B_9_ ribbon.

**Figure 3:**
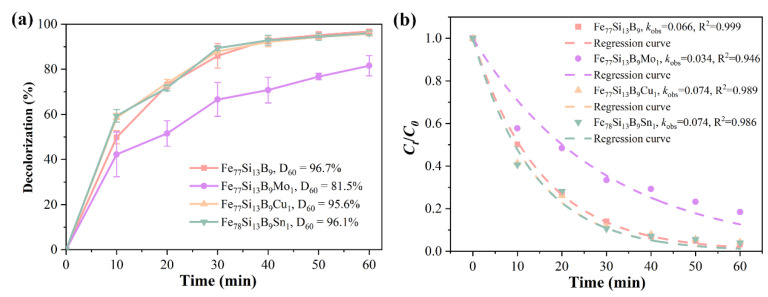
(**a**) Decolorization efficiency and (**b**) kinetic fitting (*k*_oks_ and R^2^) of *C_t_/C*_0_ for Orange II azo dye degradation by amorphous alloys with different compositions.

According to the Lambert–Beer law, the ratio of Orange II concentrations at different reaction times is directly proportional to the ratio of the intensities at the maximum absorption peak (λ_max_) [31]. Therefore, the degradation efficiency of Orange II at various reaction times, treated with different alloy ribbons, can be calculated using Equation (3), as shown in Figure 3a. Under identical reaction conditions, the decolorization process initiated more rapidly with Fe_7__7_Si_1__3_B_9_Cu_1_ and Fe_77_Si_13_B_9_Sn_1_ ribbons, achieving decolorization efficiencies of 58.5% and 59.4%, respectively, at 10 min. In contrast, the Fe_78_Si_13_B_9_ ribbon exhibited a decolorization efficiency of only approximately 50% at the same time point. Over a 60-min period, the Fe_78_Si_13_B_9_, Fe_7__7_Si_1__3_B_9_Cu_1_, and Fe_77_Si_13_B_9_Sn_1_ ribbons all achieved decolorization efficiencies of around 96%, outperforming the Fe_77_Si_13_B_9_Mo_1_ ribbon, which reached only 81.5% efficiency at 60 min.

D = [(*C*_0_ − *C_t_*)/*C*_0_] × 100 (3)

Here, D represents the decolorization efficiency, *C*_t_ is the concentration of the Orange II solution at time *t*, and *C*_0_ is the initial concentration of the Orange II solution.

To investigate the degradation rates of the three materials, the experimental data obtained from the degradation process were fitted using a pseudo-first-order kinetic model. The pseudo-first-order kinetic equation has been widely applied in the degradation of azo dyes by zero-valent metals and is expressed as follows [32]:

(4) $$C_{t}/C_{0}\;=\exp\;(-k_{\mathrm{obs}}t)$$

where *k*_obs_ is the apparent rate constant (min^−1^), and *t* is the reaction time.

Figure 3b shows the (*C_t_/C*_0_) vs. reaction time curves for the four amorphous alloy compositions. The fitted *k*_obs_ for Fe_78_Si_13_B_9_, Fe_77_Si_13_B_9_Mo_1_, Fe_77_Si_13_B_9_Cu_1_, and Fe_77_Si_13_B_9_Sn_1_ ribbons were determined to be 0.066 min^−1^, 0.034 min^−1^, 0.074 min^−1^, and 0.074 min^−1^, respectively. Compared to the *k*_oks_ of the Fe_78_Si_13_B_9_ ribbon, the Fe_77_Si_13_B_9_Cu_1_ and Fe_77_Si_13_B_9_Sn_1_ ribbons exhibited an approximately 12% increase in *k*_oks_, indicating that the addition of Cu and Sn elements effectively enhanced the decolorization performance of FeSiB amorphous alloys toward Orange II. In contrast, the incorporation of Mo hindered the decolorization process of FeSiB amorphous alloys for Orange II.

**Figure 4:**
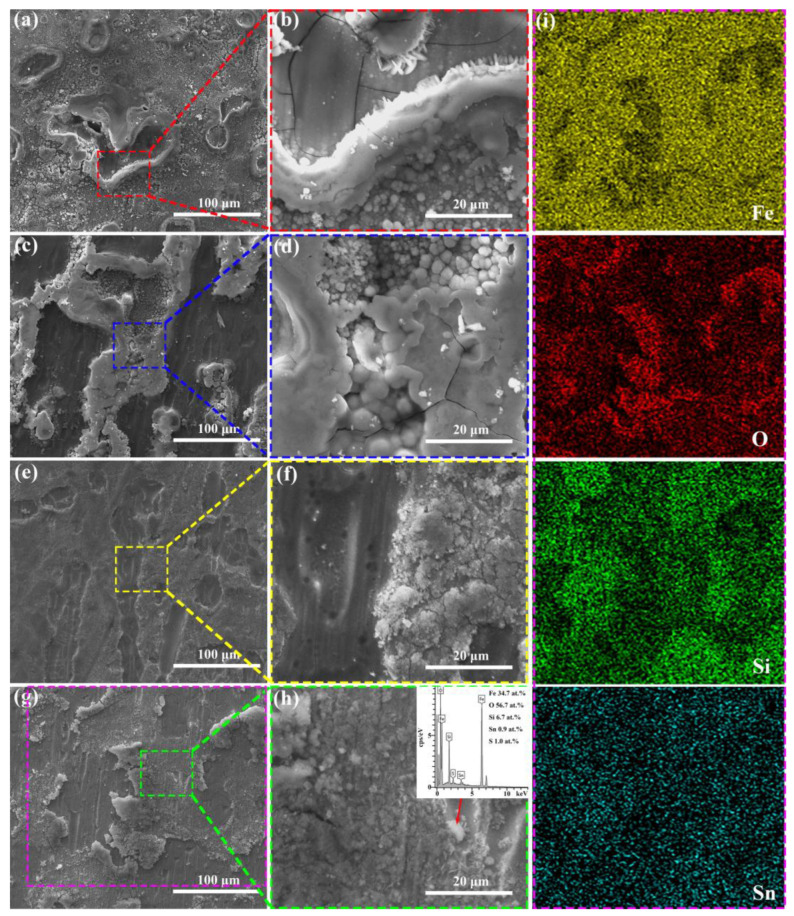
SEM images of the amorphous alloy ribbons after the degradation reaction: (**a**,**b**) Fe_78_Si_13_B_9_, (**c**,**d**) Fe_77_Si_13_B_9_Mo_1_, (**e**,**f**) Fe_7__7_Si_1__3_B_9_Cu_1_, and (**g**,**h**) Fe_77_Si_13_B_9_Sn_1_; figure (**i**) displays the EDS elemental mapping corresponding to the region shown in figure (**g**).

Since the decolorization process of Orange II azo dye by Fe-based amorphous ribbons involves a series of heterogeneous reactions on their surfaces, the surface morphology of the ribbons after reaction was analyzed. As clearly shown in Figure 4, the surfaces of all Fe-based amorphous ribbons exhibit typical characteristics of selective corrosion. Specifically, the Fe_78_Si_13_B_9_ and Fe_77_Si_13_B_9_Mo_1_ ribbons show a significant amount of granular corrosion products on their surfaces after the reaction. However, the granular corrosion products on the Fe_78_Si_13_B_9_ ribbon are more widely distributed, with three-dimensional (3D) petal-like structures surrounding the selective corrosion regions. These structures possess a high specific surface area, enabling more effective adsorption of Orange II molecules [10,25]. In contrast, the granular corrosion products on the Fe_77_Si_13_B_9_Mo_1_ ribbon are primarily concentrated in the selective corrosion regions. Compared to other amorphous alloys with different compositions, the surface outside the selective corrosion zone of Fe_77_Si_13_B_9_Mo_1_ is smoother. This may be attributed to the addition of Mo, which enhances the corrosion resistance of the amorphous alloy [33]. On the other hand, the Fe_7__7_Si_1__3_B_9_Cu_1_ and Fe_77_Si_13_B_9_Sn_1_ ribbons exhibit a large number of cotton-like deposits on their surfaces. These deposits are loose and porous, providing channels for mass and electron transfer during the reaction, which facilitates the diffusion and adsorption of Orange II dye molecules [34]. The surfaces surrounding the deposits feature 3D petal-like nanostructured corrosion products. Unlike the Fe_78_Si_13_B_9_ ribbon, the Fe_7__7_Si_1__3_B_9_Cu_1_ and Fe_77_Si_13_B_9_Sn_1_ ribbons display a higher quantity of these petal-like nanostructures, predominantly distributed in non-selective corrosion regions. Consequently, the Fe_7__7_Si_1__3_B_9_Cu_1_ and Fe_77_Si_13_B_9_Sn_1_ ribbons demonstrate superior Orange II dye removal performance, while the Fe_77_Si_13_B_9_Mo_1_ ribbon, lacking such structures, exhibits the poorest dye removal performance among the four types of ribbons. Additionally, EDS analysis of the deposits in Figure 4h reveals the presence of sulfur (S) elements. Since the ribbons themselves do not contain S, its presence indicates that azo dye molecules or their degradation intermediates/products are adsorbed on the ribbon surfaces [35]. Figure 4i presents the EDS elemental mapping corresponding to the location depicted in Figure 4g. It is evident that the selectively corroded regions exhibit higher concentrations of Fe, Si, and Sn, whereas the surrounding areas display an elevated oxygen (O) content, indicating that iron hydroxides are predominantly distributed outside the selectively corroded regions.

**Figure 5:**
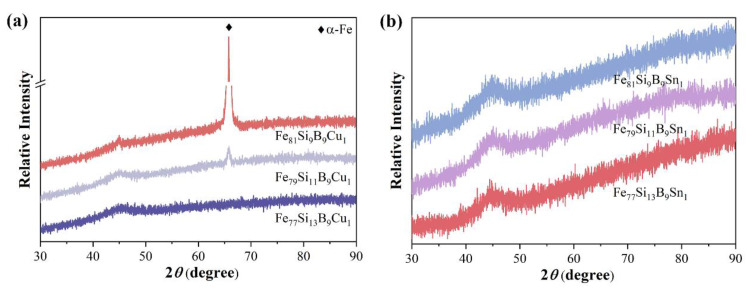
XRD patterns of (**a**) Fe_77+x_Si_13−x_B_9_Cu_1_ (x = 0, 2, or 4) and (**b**) Fe_77+x_Si_13−x_B_9_Sn_1_ (x = 0, 2, or 4).

Figure 5a presents the XRD patterns of Fe_77+x_Si_13−x_B_9_Cu_1_ (x = 0, 2, or 4). As Fe substitutes for Si, the Fe_7__9_Si_1__1_B_9_Cu_1_ alloy ribbon begins to exhibit a distinct diffraction peak near 65°, corresponding to the α-Fe phase. With further increases in Fe content, the intensity of the diffraction peak significantly increases, indicating enhanced crystallinity of the alloy. These results demonstrate that the substitution of Fe for Si reduces the glass-forming ability of Fe_77+x_Si_13−x_B_9_Cu_1_ (x = 0, 2, or 4) alloys, leading to the formation of α-Fe crystalline phases starting from the Fe_79_Si_11_B_9_Cu_1_ alloy. Figure 5b shows the XRD patterns of Fe_77+x_Si_13−x_B_9_Sn_1_ (x = 0, 2, or 4). It is evident that all alloys exhibit a typical broad diffuse scattering peak at 2*θ* = 45° without any crystalline diffraction peaks, confirming the fully amorphous structure of the prepared samples. These results indicate that, within the FeSiB alloy system, the addition of Sn enhances the glass-forming ability more effectively than the addition of Cu. Table 1 summarizes the elemental composition of the Fe_77+x_Si_13−x_B_9_Cu_1_ (x = 0, 2, or 4) and Fe_77+x_Si_13−x_B_9_Sn_1_ (x = 0, 2, or 4) ribbons measured by EDS, which generally aligns with the nominal compositional trends.

**Figure 6:**
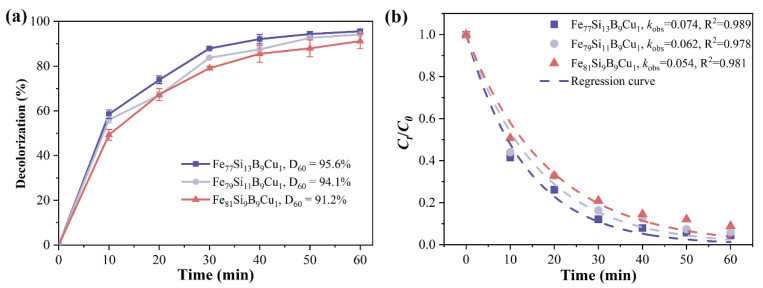
(**a**) Decolorization efficiency and (**b**) kinetic fitting (*k*_oks_ and R^2^) of *C_t_/C*_0_ for Orange II azo dye degradation by Fe_77+x_Si_13−x_B_9_Cu_1_(x = 0, 2 or 4) alloys.

Figure 6a shows the time-dependent decolorization efficiency of Fe_77+x_Si_13−x_B_9_Cu_1_ (x = 0, 2, or 4) alloys for Orange II. Under identical conditions, the decolorization efficiency of Fe_77+x_Si_13−x_B_9_Cu_1_ alloys gradually decreases with increasing Fe content. At 60 min of reaction, the decolorization efficiencies of Fe_7__9_Si_1__1_B_9_Cu_1_ and Fe_8__1_Si_9_B_9_Cu_1_ alloys for Orange II are 94.1% and 91.2%, respectively, both lower than that of Fe_7__7_Si_1__3_B_9_Cu_1_ (95.6%). Figure 6b presents the *k*_oks_ and R^2^ for the degradation reactions of Fe_77+x_Si_13−x_B_9_Cu_1_ (x = 0, 2, or 4) alloys. It can be observed that the degradation rate constants of Fe_7__9_Si_1__1_B_9_Cu_1_ and Fe_8__1_Si_9_B_9_Cu_1_ alloys for Orange II azo dye are 0.062 min^−1^ and 0.054 min^−1^, respectively, representing reductions of 16.2% and 27.0% compared to that of Fe_7__7_Si_1__3_B_9_Cu_1_ (0.074 min^−1^). Previous studies have shown that the reactivity of amorphous Fe^0^ in Fe-based alloys of the same composition is higher than that of crystalline Fe^0^. Therefore, the amorphous Fe_7__7_Si_1__3_B_9_Cu_1_ exhibits superior decolorization performance for Orange II compared to the partially crystallized Fe_7__9_Si_1__1_B_9_Cu_1_ and Fe_8__1_Si_9_B_9_Cu_1_ alloys, and the decolorization performance deteriorates progressively with increasing crystallinity.

**Figure 7:**
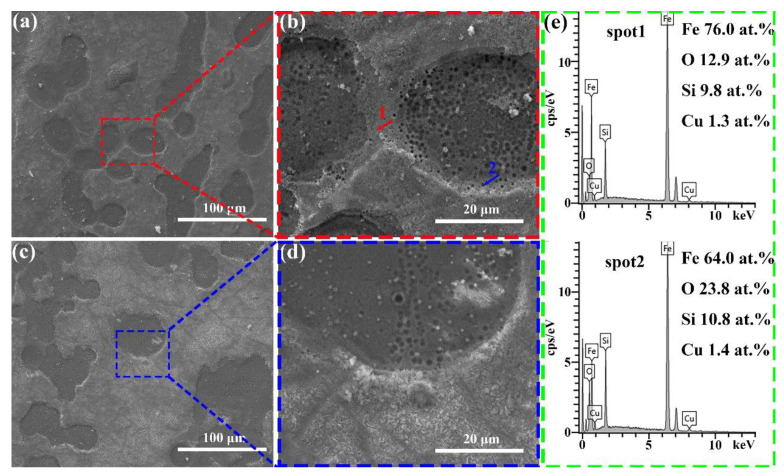
(**a**,**b**) Fe_7__9_Si_1__1_B_9_Cu_1_ alloy ribbons and (**c**,**d**) Fe_8__1_Si_9_B_9_Cu_1_ alloy ribbons after the decolorization reaction; (**e**) EDS point scan of the region marked in (**b**).

Figure 7a–d present the surface morphology of the Fe_7__9_Si_1__1_B_9_Cu_1_ and Fe_8__1_Si_9_B_9_Cu_1_ alloy ribbons after the reaction. Compared to the amorphous Fe_7__7_Si_1__3_B_9_Cu_1_, the Fe_7__9_Si_1__1_B_9_Cu_1_ and Fe_8__1_Si_9_B_9_Cu_1_ alloys exhibit numerous nanoscale pores within the selective corrosion regions, which are likely formed due to the detachment of α-Fe during the decolorization of Orange II [36]. In addition, the Fe_7__7_Si_1__3_B_9_Cu_1_ amorphous ribbon exhibited significantly more cotton-like deposits on its surface after the reaction compared to the Fe_7__9_Si_1__1_B_9_Cu_1_ and Fe_8__1_Si_9_B_9_Cu_1_ alloy ribbons, indicating that more Fe^0^ participated in the reaction during the decolorization process for Fe_7__7_Si_1__3_B_9_Cu_1_. The EDS data in Figure 7e reveal that the Fe content in the pore-free regions of the selective corrosion zones is 76%, significantly higher than the 64% observed in the nanoporous regions.

**Figure 8:**
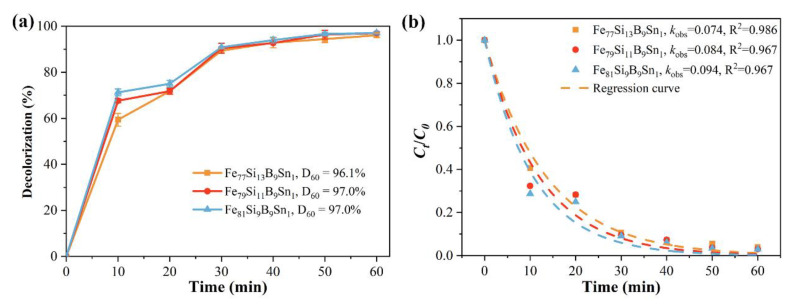
(**a**) Decolorization efficiency and (**b**) kinetic fitting (*k*_oks_ and R^2^) of *C_t_/C*_0_ for Orange II azo dye degradation by Fe_77+x_Si_13−x_B_9_Sn_1_ (x = 0, 2 or 4) alloys.

Figure 8a shows the time-dependent decolorization efficiency of Fe_77+x_Si_13−x_B_9_Sn_1_ (x = 0, 2, or 4) alloys for Orange II. Under identical conditions, the decolorization efficiency of Fe_77+x_Si_13−x_B_9_Sn_1_ alloys gradually increases with higher Fe content. At 60 min of reaction, the decolorization efficiencies of Fe_79_Si_11_B_9_Sn_1_ and Fe_81_Si_9_B_9_Sn_1_ amorphous ribbons for Orange II both reach 97.0%, surpassing that of Fe_77_Si_13_B_9_Sn_1_ (96.1%). Notably, the Fe_81_Si_9_B_9_Sn_1_ ribbon achieves a decolorization efficiency of 71.3% within just 10 min. Figure 8b presents the *k*_oks_ and the R^2^ for the degradation reactions of Fe_77+x_Si_13−x_B_9_Sn_1_ (x = 0, 2, or 4) alloys. It can be observed that the degradation rate constants of Fe_79_Si_11_B_9_Sn_1_ and Fe_81_Si_9_B_9_Sn_1_ alloys for Orange II azo dye are 0.084 min^−1^ and 0.094 min^−1^, respectively, representing improvements of 13.5% and 27.0% compared to that of Fe_77_Si_13_B_9_Sn_1_ (0.074 min^−1^). These results demonstrate that, while maintaining the amorphous structure of the ribbons, increasing the Fe content effectively enhances the decolorization performance of FeSiBSn amorphous alloys for azo dyes. Table 2 shows the removal performance of certain Fe-based amorphous alloys for azo dyes. A comparison reveals that the Fe_81_Si_9_B_9_Sn_1_ amorphous ribbon exhibits exceptional removal performance under neutral conditions, which can even rival the removal performance of some compositions at pH = 3.

**Figure 9:**
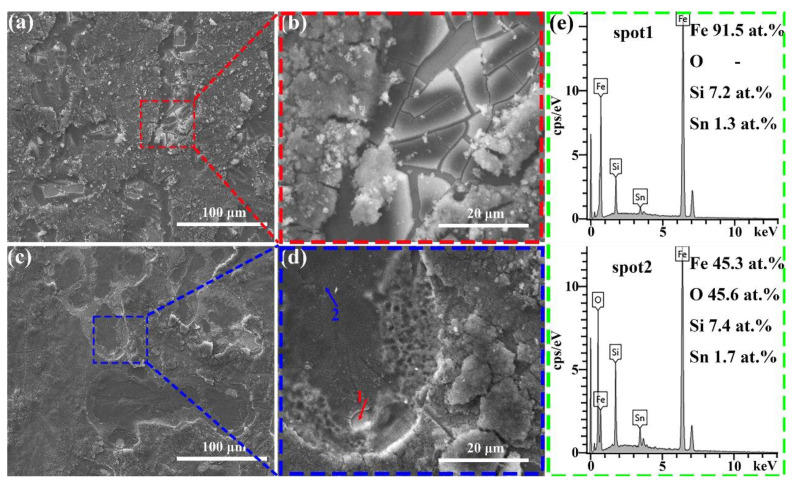
(**a**,**b**) Fe_79_Si_11_B_9_Sn_1_ alloy ribbons and (**c**,**d**) Fe_81_Si_9_B_9_Sn_1_ alloy ribbons after the decolorization reaction; (**e**) EDS point scan of the region marked in (**d**).

Figure 9a–d present the surface morphology of Fe_79_Si_11_B_9_Sn_1_ and Fe_81_Si_9_B_9_Sn_1_ amorphous ribbons after the reaction. With increasing Fe content, the Fe_77+x_Si_13−x_B_9_Sn_1_ (x = 0, 2, or 4) alloys exhibit numerous cracks on their surfaces after the reaction, with fresh ribbon surfaces exposed within the cracks. As the degradation reaction progresses, these cracks gradually peel off, continuously exposing fresh internal surfaces and providing a sustained supply of Fe^0^ for the degradation process. The EDS data in Figure 9e reveal that the Fe content in the regions where cracks have peeled off is 91.5%, nearly identical to the Fe content on the ribbon surface before the reaction (as shown in Table 1). Additionally, the absence of O indicates that these regions remain unoxidized. This self-renewing behavior endows the FeSiBSn amorphous ribbons with exceptional decolorization performance for azo dyes.

**Figure 10:**
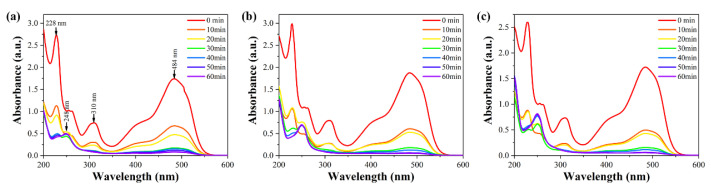
UV-Vis absorption spectra of Orange II solutions at different reaction times for (**a**) Fe_77_Si_13_B_9_Sn_1_, (**b**) Fe_79_Si_11_B_9_Sn_1_, and (**c**) Fe_81_Si_9_B_9_Sn_1_ amorphous ribbons.

The UV-Vis absorption spectra of Orange II solutions at different reaction times for Fe_77_Si_13_B_9_Sn_1_, Fe_79_Si_11_B_9_Sn_1_, and Fe_81_Si_9_B_9_Sn_1_ amorphous ribbons are shown in Figure 10. From the 0-min curve, it can be observed that Orange II solutions exhibit three characteristic absorption peaks at 484 nm, 310 nm, and 228 nm. Among them, the λ_max_ at 484 nm originates from the n-π* transition of the azo structure (-N=N-), while the peaks at 228 nm and 310 nm are attributed to the π-π* transitions of the conjugated systems of the benzene and naphthalene rings, respectively [39]. The concentration of Orange II solution is proportional to the intensity at λ_max_. Therefore, the degradation efficiency of the amorphous alloys for Orange II dye can be represented by the intensity changes of the absorption peak at 484 nm. The results indicate that the intensity of the absorption peak at 484 nm gradually decreases with increasing reaction time, nearly disappearing after 60 min, suggesting that the chromophore (-N=N-) in the dye molecules is almost completely broken. Additionally, the characteristic absorption peaks at 228 nm and 310 nm disappear after 30 min of reaction, indicating that the benzene and naphthalene ring structures in the dye molecules are also degraded [27,40]. At this stage, a new characteristic absorption peak appears at 248 nm, corresponding to the amino structure (-NH_2_), which is identified as a product of the cleavage of the azo bond. In the initial stages of the decolorization process, the decolorization of azo dyes by FeSiBSn is dominated by surface adsorption, and thus no new characteristic peaks are observed. By comparing the data in Figure 10, it is evident that the intensity of the amino characteristic peak increases with higher Fe content, indicating that the degradation efficiency for Orange II follows the order: Fe_81_Si_9_B_9_Sn_1_ > Fe_79_Si_11_B_9_Sn_1_ > Fe_77_Si_13_B_9_Sn_1_.

## 4. Conclusions

This study compares the decolorization performance of Fe_78_Si_13_B_9_ and Fe_77_Si_13_B_9_M_1_ (M = Mo, Cu, or Sn) amorphous alloys for azo dyes, as well as the influence of Fe content variation on the decolorization performance of Fe_77+x_Si_13−x_B_9_Cu_1_ (x = 0, 2, or 4) and Fe_77+x_Si_13−x_B_9_Sn_1_ (x = 0, 2, or 4) alloys. For the Fe_77_Si_13_B_9_M_1_ amorphous alloys, the addition of Cu and Sn elements enhances their decolorization performance for azo dyes, while the incorporation of Mo has a detrimental effect. The underlying mechanisms can be primarily attributed to the following aspects: The incorporation of Mo enhances the corrosion resistance of amorphous alloys, consequently delaying the surface reaction of Fe^0^ and reducing the reactive activity at the alloy surface. In contrast, the addition of Cu and Sn induces the formation of abundant 3D petal-like nanostructures corrosion products during surface reactions. These unique nanostructures significantly enhance the adsorption capacity of amorphous alloys toward azo dye molecules, thereby facilitating the decolorization process through improved interfacial interactions. In the Fe_77+x_Si_13−x_B_9_Cu_1_ alloys, increasing Fe content reduces the glass-forming ability, leading to the precipitation of α-Fe crystalline phases, thereby weakening the decolorization performance of FeSiBCu alloys for azo dyes. In contrast, the Fe_77+x_Si_13−x_B_9_Sn_1_ alloys exhibit excellent glass-forming ability, maintaining their amorphous structure even with increasing Fe content, and their decolorization performance for azo dyes improves accordingly. The outstanding decolorization performance of Fe_77+x_Si_13−x_B_9_Sn_1_ amorphous alloys for Orange II are attributed to the formation of numerous cracks during the reaction, which peel off to expose fresh surfaces, enabling a self-renewing behavior. This self-renewing behavior results in a degradation rate of 0.094 min^−1^ for Fe_81_Si_9_B_9_Sn_1_, significantly higher than that of the conventional Fe_78_Si_13_B_9_ amorphous alloy (0.066 min^−1^). This study provides a new perspective for designing FeSiB amorphous alloys with high-efficiency decolorization performance for azo dyes.

## Figures and Tables

**Table 1:** Elemental composition (at.%) of the Fe_77+x_Si_13−x_B_9_Cu_1_ (x = 0, 2, or 4) and Fe_77+x_Si_13−x_B_9_Sn_1_ (x = 0, 2, or 4) ribbons measured by EDS.

Sample	Fe	Si	Cu	Sn
Fe_77_Si_13_B_9_Cu_1_	86.8	11.6	1.6	-
Fe_79_Si_11_B_9_Cu_1_	89.0	9.7	1.3	-
Fe_81_Si_9_B_9_Cu_1_	90.8	7.9	1.3	-
Fe_77_Si_13_B_9_Sn_1_	87.3	11.5	-	1.2
Fe_79_Si_11_B_9_Sn_1_	89.3	9.4	-	1.3
Fe_81_Si_9_B_9_Sn_1_	91.5	7.3	-	1.2

**Table 2:** Part of research on azo dye using Fe-base amorphous alloys.

Composition	Dye	*C*_0_ (mg/L)	pH	Mass Dosage (g/L)	Temp. (°C)	Kobs (min^−1^)	Ref.
Fe_78_(Si,B)_22_	Orange II	100	6	9-12	Room	0.125	[37]
(Fe_0.99_Mo_0.01_)_78_Si_9_B_13_	Direct blue 2B	200	7	13.3	60	0.136	[38]
Fe_78_Si_13_B_9_	Orange II	40	7	2	35	0.071	[11]
Fe_80_B_13_C_7_	acid orange 7	20	3	2	25	0.08	[10]
Fe_85_P_11_C_2_B_2_	acid orange 7	20	3	2	35	0.0118	[12]
Fe_78_Si_11_B_9_P_2_	Orange II	40	7	2	35	0.082	[27]
Fe_81_Si_9_B_9_Sn_1_	Orange II	40	7	2	35	0.094	This work

## Data Availability

The original contributions presented in this study are included in the article. Further inquiries can be directed to the corresponding authors.
